# Association between the triglyceride-glucose index and the presence and prognosis of coronary microvascular dysfunction in patients with chronic coronary syndrome

**DOI:** 10.1186/s12933-023-01846-z

**Published:** 2023-05-13

**Authors:** Wen Zhang, Lu Liu, Huiying Chen, Siqi Li, Minying Wan, Abdul-Quddus Mohammed, Bin Xu, Guoqing Yin, Xian Lv, Tingting Shi, Jassur Galip, Ayman A. Mohammed, Redhwan M. Mareai, Yawei Xu, Fuad A. Abdu, Wenliang Che

**Affiliations:** 1 Department of Cardiology, Shanghai Tenth People’s Hospital, Tongji University School of Medicine, 301 Yanchang Road, Shanghai, 200072 China; 2 Department of Cardiology, Shanghai Tenth People’s Hospital Chongming Branch, Tongji University School of Medicine, 301 Yanchang Road, Shanghai, 200072 China

**Keywords:** Chronic coronary syndrome, Coronary microvascular dysfunction, Coronary angiography‑derived index of microvascular resistance, Triglyceride-glucose index, Clinical outcomes

## Abstract

**Background:**

Coronary microvascular dysfunction (CMD) is a strong determinant of prognosis in patients with chronic coronary syndrome (CCS). The triglyceride-glucose index (TyG index), an alternative method to evaluate insulin resistance, is positively correlated with the incidence and adverse outcomes of cardiovascular diseases. However, the relationship between the TyG index and the presence and prognosis of CMD in CCS patients has not been investigated. Therefore, we aimed to evaluate the association between the TyG index and the presence and clinical outcomes of CMD among CCS patients.

**Methods:**

CCS patients who underwent coronary angiography between June 2015 to June 2019 were included. The TyG index was calculated as Ln[fasting triglycerides (mg/dL) × fasting blood glucose (mg/dL)/2]. Coronary angiography‑derived index of microvascular resistance (caIMR) was used to measure microvascular function, and CMD was defined as caIMR ≥ 25U. Patients with CMD were divided into three groups (T1, T2, and T3 groups) according to TyG tertiles. The primary endpoint was major adverse cardiac event (MACE).

**Results:**

Of 430 CCS patients, 221 patients had CMD. CMD patients had significantly higher TyG index than those without CMD. Sixty-three MACE was recorded during the follow-up duration among CMD patients, and the incidence rate of MACE was higher in the T3 group compared to T1/T2 groups (39.2% vs. 20.5% vs. 25.7%; P = 0.035). Multivariable logistic regression analysis showed that the TyG index was an independent predictor of CMD (OR, 1.436; 95% CI, 1.014–2.034; P = 0.042). Compared to the T1 group, the T3 group strongly correlated with the risk of MACE in CMD patients even after adjusting for additional confounding risk factors (HR, 2.132; 95%CI, 1.066–4.261; P = 0.032).

**Conclusion:**

TyG index is significantly associated with the risk of CMD, and it is an independent predictor of MACE among CMD patients with CCS. This study suggests that the TyG index has important clinical significance for the early prevention and risk stratification of CMD.

**Supplementary Information:**

The online version contains supplementary material available at 10.1186/s12933-023-01846-z.

## Background

Chronic coronary syndrome (CCS) is a clinical manifestation of coronary artery disease (CAD) other than acute coronary thrombosis [1]. Coronary microvascular dysfunction (CMD) is a clinical situation where the structure or function of coronary micro-vessels is affected, which is widely present in CCS patients, and may interact with obstructive atherosclerosis to accelerate the progression of the cardiovascular disease (CVD) [2–4]. Numerous clinical investigations have demonstrated that CMD is linked to poor prognosis in a spectrum of CVD such as acute coronary syndrome (ACS), heart failure with preserved ejection fraction (HFpEF), myocardial infarction with nonobstructive coronary arteries (MINOCA) and CCS [5–9], posing a severe threat to people's health. Therefore, early prevention and risk stratification of CMD is crucial in order to avoid further progression and enhance the quality of life.

Insulin resistance (IR) refers to the decrease in insulin sensitivity that requires an increase in the amount of insulin to achieve its normal function. It has been proven to be one of the major risk factors, which is closely related to the occurrence and development of CMD [10, 11]. However, challenges in implementing existing detection methods for IR in clinical practice and a lack of studies investigating its prognostic value in CMD patients underscore the need for further research. The triglyceride-glucose index (TyG index) based on fasting blood glucose (FBG) and fasting triglycerides (TG) has been proposed as an alternative method to evaluate IR [12, 13]. Several studies have found a positive correlation between the TyG index and the presence and poor clinical outcomes among CVD in diabetic or nondiabetic patients, including carotid atherosclerosis, CCS, arterial stiffness, MINOCA, premature coronary artery disease, ACS and coronary chronic total occlusion [14–17]. Moreover, recent research has shown that the TyG index was independently correlated with the likelihood of coronary slow flow, suggesting that the TyG index may be related to the presence of CMD [18]. However, the association between the TyG index and the presence and prognosis of CMD in patients with CCS has not been studied before.

Hence, this study aimed to explore the association between the TyG index and the risk of CMD in CCS patients and to evaluate the prognostic value of the TyG index among CMD patients with CCS.

## Methods

### Study population

This was a single-center retrospective observational study that recruited all consecutive patients admitted for CCS who underwent coronary angiography (CAG) at Shanghai Tenth People's Hospital from June 2015 to June 2019.

In the present study, we included patients aged > 18 years with suspected or established CCS, diagnosed according to the 2019 ESC guidelines for the diagnosis and management of CCS [1]. The major exclusion criteria included myocardial infarction (MI) within 7 days, post coronary artery bypass graft surgery (CABG), severe hepatic or renal insufficiency, malignancy, left ventricular ejection fraction (LVEF) < 35%, or other severe medical illnesses. The angiography images with low contrast opacification, obvious vascular overlap/distortion of the interrogated artery, or poor quality were excluded according to the previous study [19].

Clinical data of all patients were collected from medical files by trained physicians who were blinded to the research protocol. Venous blood samples were obtained after an overnight fast on admission to analyze FBG, hemoglobin A1c (HbA1c), total cholesterol (TC), TG, low-density lipoprotein-C (LDL-C), serum creatinine, C-reactive protein (CRP) levels. The data of echocardiography and CAG were obtained from examination report sheets.

Our study protocol was conducted in accordance with the Helsinki Declaration and was approved by the ethical review board of Shanghai Tenth People’s Hospital. Written informed consent was obtained from each patient who was enrolled in the study.

### Evaluation of coronary microvascular function

The coronary microvascular function was assessed on all participants with the coronary angiography-derived index of microcirculatory resistance (caIMR) measurement. The caIMR analysis was performed by trained readers who were unaware of outcome data in the Cath lab of the Shanghai Tenth People's Hospital using the FlashAngio system package (FlashAngio, Rainmed Ltd., Suzhou, China). Previous studies have described the detailed theories of caIMR measurement, which was calculated using the following formula [19]:

$$ {\text{calMR}} = \left( {Pd} \right){}_{hyp}\frac{L}{{V_{diastole} }} $$

where (Pd)_hyp_ is the mean pressure (unit: mmHg) at the distal position of the coronary artery at the maximal hyperemia, L is a constant (L = 75), which mimics the length from the inlet of the coronary arterial tree to the distal position, and V_diastole_ is the mean blood flow velocity (unit: mm/s) at the distal position of the coronary artery at diastole, and V_hyp_ = K·V_diastole_ (K = 2.1) is the mean blood flow velocity (unit: mm/s) at the distal position of the coronary artery at the maximal hyperemia.

Microvascular functions were measured in 642 coronary arteries among 430 CCS patients. caIMR was measured primarily in stenotic epicardial arteries, and for those patients without significant coronary stenosis, we evaluated caIMR in all measurable epicardial arteries. Percutaneous coronary intervention (PCI) was performed when there was significant evidence of myocardial ischemia and the degree of coronary stenosis was greater than 70%. Patients who underwent PCI had their caIMR measured after PCI. The vessel with the highest value of caIMR among the coronary arteries of the patient was chosen.

### Definitions and grouping

DM patients were diagnosed if they met one of the following: (1) HbA1c ≥ 6.5%; (2) Random plasma glucose ≥ 11.1 mmol/L; (3) Fasting plasma glucose ≥ 7.0 mmol/L; and (4) 2‑hour glucose in venous plasma ≥ 11.1 mmol/L according to OGTT[20]. Hypertension was defined as systolic blood pressure (SBP) ≥ 140 mmHg or diastolic blood pressure (DBP) ≥ 90 mmHg at rest or receiving antihypertensive treatments. Body mass index (BMI) was calculated according to the following formula: BMI (kg/m^2^) = weight (kg) /height^2^ (m^2^).

Based on the cut-off value previously established, CMD was defined as caIMR ≥ 25U [19]. The TyG index was computed as Ln[fasting TG (mg/dL) × FBG (mg/dL)/2] [12]. Patients with CMD were divided into three groups (T1, T2, and T3 groups) according to the TyG tertiles.

### Follow-up and endpoint of the study

The median follow-up period in our study was 35 months. Follow-up data was performed by trained physicians at Shanghai Tenth People's Hospital via telephone calls, hospital records, and outpatient visits. The primary endpoint of this study was major adverse cardiac events (MACE), which was a composite of cardiovascular death, nonfatal MI, heart failure, nonfatal stroke, and ischemia-driven revascularization. Cardiovascular death was defined as death due to malignant arrhythmia, acute MI, heart failure, or other cardiac diseases. Nonfatal MI was defined as positive cardiac biomarkers with the typical symptoms of myocardial ischemia or dynamic changes in electrocardiograms [21]. Diagnosis of heart failure was based on previous guidelines [22]. Nonfatal stroke referred to acute cerebral infarction diagnosed by typical clinical symptoms or imaging examination [23]. Ischemia-driven revascularization was the revascularization as a result of recurrent angina or a positive test for cardiac ischemia.

### Statistical analysis

The data of our study were analyzed using the Statistical Package for Social Sciences (SPSS) v.22., and the figures were drawn by GraphPad softwarev.8.0.1. Continuous variables were presented as the mean ± standard deviation in the case of normal distribution, and medians (interquartile range) in the case of non-normal distribution. The intergroup comparisons of continuous variables were analyzed using a t-test or ANOVA if they had a normal distribution and using Mann–Whitney U-test or Kruskal–Wallis test if they presented a non-normal distribution. Categorical variables were displayed as counts and percentages and were compared using Pearson’s chi-squared (χ^2^) test or Fisher’s exact test as the case may be. Univariate and multivariate logistic regression analyses were used to analyze the correlation between clinical risk factors and CMD patients. Pearson or Spearman correlation analysis was applied to determine the correlation between the TyG index and caIMR. Kaplan–Meier survival curves were used to evaluate the MACE-free survival rates, and discrepancies in survival rates were compared using the log-rank test. The association between the TyG index and outcomes in CMD patients was determined using univariate and multivariate Cox proportional regression models. In the logistic regression and Cox regression, univariate predictors with P < 0.10 were included for multivariable models. The collinearity diagnosis was used to assess collinearity between the variables included in the multivariable models. When variance inflation factor (VIF) was less than 10, we considered that no evidence of model collinearity was found. We excluded those variables that are highly correlated when performing multivariate regression analysis. We conducted subgroup analysis to test whether the correlation between TyG index and the presence and prognosis of CMD differed across subgroups and *p* for interaction was calculated. All analysis was conducted two-sided, and statistical significance was set at P-value < 0.05.

## Results

### Baseline characteristics

**Fig. 1:**
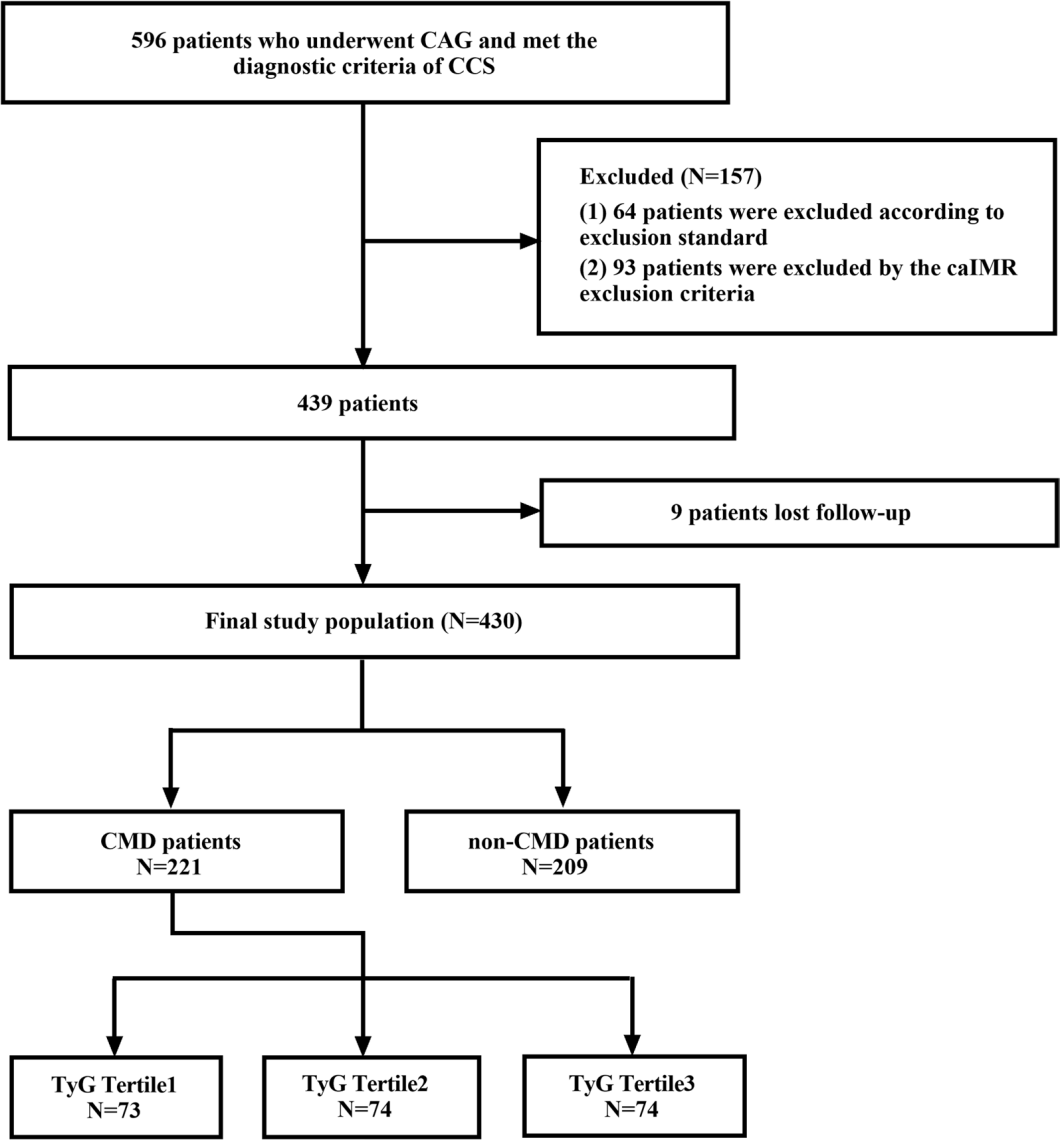
Flow diagram of study participants. *CAG* coronary angiography, *CCS* chronic coronary syndrome, *caIMR* coronary angiography-derived index of microcirculatory resistance, *CMD* coronary microvascular dysfunction, *TyG index* triglyceride-glucose index.

A total of 596 patients who underwent CAG and met the diagnostic criteria of CCS were included, in which 64 patients were excluded according to the exclusion criteria, 93 patients were excluded due to the caIMR exclusion criteria, and 9 patients were lost to follow-up. Four hundred and thirty patients were finally included in the present study (Fig. 1).

**Fig. 2:**
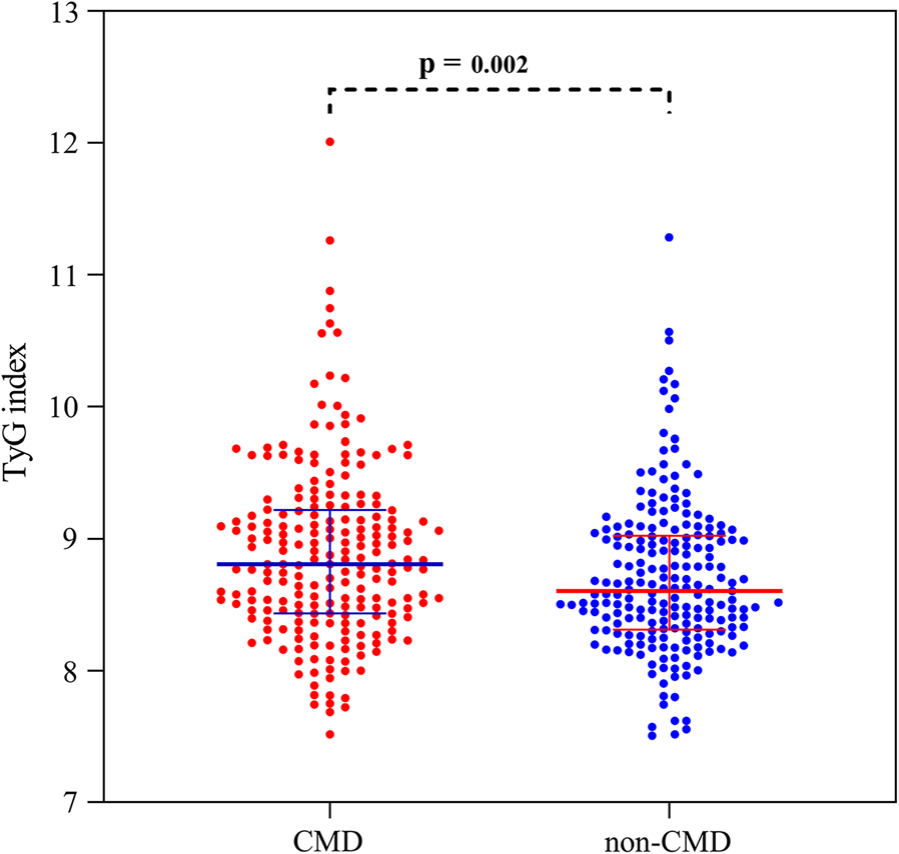
The distribution of TyG index at CMD and non-CMD patients. *TyG index* triglyceride-glucose index, *CMD* coronary microvascular dysfunction.

Population characteristics of CCS patients were shown in Table 1. Of 430 CCS patients, 221 patients had CMD. CMD patients had a significantly higher TyG index than those without CMD [8.81(8.43,9.22) vs. 8.60(8.31,9.02); P = 0.002] (Table 1; Fig. 2). Moreover, caIMR, caFFR, and BMI were also significantly higher in CMD patients than in non-CMD patients. Laboratory findings also showed that the FBG, TG, and TC were higher in the CMD patients. However, the history of CAD, 3-vessel disease, PCI and clopidogrel use was lower in the CMD group than in the non-CMD group.

**Table 1:** Clinical characteristics of the study population stratified by CMD

	Total patients (N = 430)	CMD (N = 221)	Non-CMD (N = 209)	P value
General characteristics				
Age (years)	63.54 ± 9.61	63.12 ± 9.13	63.99 ± 10.09	0.352
Male, n (%)	254 (59.1)	130 (58.8)	124 (59.3)	0.915
BMI (kg/m^2^)	24.99 ± 3.16	25.56 ± 3.25	24.39 ± 2.96	< 0.001
Heart rate (beats per minute)	76.46 ± 11.41	76.65 ± 11.28	76.25 ± 11.57	0.715
Comorbidities				
Atrial fibrillation, n (%)	22 (5.1)	9 (4.1)	13 (6.2)	0.312
Smoking, n (%)	86 (20.0)	44 (19.9)	42 (20.1)	0.962
Hypertension, n (%)	271(63.0)	135 (61.1)	136 (65.1)	0.392
PCI performed, n (%)	221(51.4)	101 (45.7)	120 (57.4)	0.015
Diabetes, n (%)	150 (34.9)	84 (38.0)	66 (31.6)	0.162
CKD, n (%)	31 (7.2)	20 (9.0)	11 (5.3)	0.129
CAD, n (%)	268 (62.3)	121 (54.8)	147 (70.3)	0.001
1- vessel disease	114 (26.5)	54 (24.4)	60 (28.7)	0.316
2- vessel disease	94 (21.9)	44 (19.9)	50 (23.9)	0.314
3- vessel disease	60 (14.0)	23 (10.4)	37 (17.7)	0.029
Laboratory values				
TyG index	8.70 (8.37,9.11)	8.81 (8.43,9.22)	8.60 (8.31,9.02)	0.002
TG (mmol/L)	1.41 (1.05,2.11)	1.51 (1.08,2.20)	1.30 (1.00,1.95)	0.012
FBG (mmol/L)	5.20 (4.80,6.10)	5.40 (4.80,6.45)	5.20 (4.70,5.80)	0.034
HbA1c (%)	6.36 ± 1.19	6.40 ± 1.08	6.31 ± 1.30	0.435
CRP (mg/L)	3.85 ± 6.77	3.95 ± 7.74	3.74 ± 5.52	0.749
Serum creatinine (umol/L)	72.40 (61.80,83.70)	72.00 (61.65,84.25)	72.70 (62.05,82.90)	0.927
TC (mmol/L)	3.58 (3.02,4.45)	3.72 (3.08,4.58)	3.50 (2.97,4.27)	0.032
LDL-C (mmol/L)	2.01 (1.50,2.68)	2.12 (1.51,2.81)	1.91 (1.50,2.39)	0.095
LVEF (%)	61.85 ± 6.21	61.91 ± 5.95	61.79 ± 6.48	0.841
Coronary physiological parameters				
caFFR	0.94 (0.91,0.96)	0.95 (0.93,0.97)	0.92 (0.90,0.95)	< 0.001
caIMR	25.25 (20.90,35.30)	35.10 (29.50,42.25)	20.80 (16.95,23.15)	< 0.001
Cardiovascular medical therapy				
Aspirin, n (%)	302 (70.2)	155 (70.1)	147 (70.3)	0.964
Clopidogrel, n (%)	241 (56.0)	109 (49.3)	132 (63.2)	0.004
Statin, n (%)	382 (88.8)	192 (86.9)	190 (90.9)	0.185
ACEI/ARB, n (%)	198 (46.0)	100 (45.2)	98 (46.9)	0.733
Beta blocker, n (%)	205 (47.7)	103 (46.6)	102 (48.8)	0.648
CCB, n (%)	166 (38.6)	88 (39.8)	78 (37.3)	0.595

*CMD* coronary microvascular dysfunction, *BMI* body mass index, *PCI* percutaneous coronary intervention, *CKD* chronic kidney disease, *CAD* coronary artery disease, *TyG index* triglyceride-glucose index, *TG* triglyceride, *FBG* fasting blood glucose, *HbA1c* hemoglobin A1c, *CRP* C-reactive protein, *TC* total cholesterol, *LDL-C* low-density lipoprotein-cholesterol, *LVEF* left ventricular ejection fraction, *caFFR* coronary angiography-derived fractional flow reserve, *caIMR* coronary angiography-derived index of microcirculatory resistance, *ACEI/ARB* angiotensin-converting-enzyme inhibitor/angiotensin receptor blocker, *CCB* calcium channel blocker

CMD patients were divided into 3 groups (T1, T2, and T3 group) based on the levels of the TyG index (Table 2). The CMD patients with higher TyG index (T3 group) tended to have atrial fibrillation and diabetes, and higher BMI, FBG, TG, TC, LDL-C, and HbA1c.

**Table 2:** Clinical characteristics of the study population stratified by TyG index

	TyG index level			P value
	T1(N = 73)	T2(N = 74)	T3(N = 74)	
General characteristics				
Age (years)	64.79 ± 9.77	61.88 ± 9.44	62.72 ± 7.97	0.138
Male, n (%)	43 (58.9)	40 (54.1)	47 (63.5)	0.505
BMI (kg/m^2^)	24.32 ± 2.96	25.83 ± 3.29	26.52 ± 3.14	< 0.001
Heart rate (beats per minute)	76.26 ± 11.30	76.42 ± 11.69	77.27 ± 10.97	0.844
Comorbidities				
Atrial fibrillation, n (%)	0	2 (2.7)	7 (9.5)	0.005
Smoking, n (%)	14 (19.2)	13 (17.6)	17 (23.0)	0.700
Hypertension, n (%)	42 (57.5)	41 (55.4)	52 (70.3)	0.134
PCI performed, n (%)	36 (49.3)	27 (36.5)	38 (51.4)	0.145
Diabetes, n (%)	17 (23.3)	30 (40.5)	37(50.0)	0.003
CKD, n (%)	6 (8.2)	7 (9.5)	7 (9.5)	0.955
CAD, n (%)	44 (60.3)	34 (45.9)	43 (58.1)	0.169
1-vessel disease	17 (23.3)	15 (20.3)	22 (29.7)	0.392
2-vessel disease	18 (24.7)	11 (14.9)	15 (20.3)	0.330
3-vessel disease	9 (12.3)	8 (10.8)	6 (8.1)	0.697
Laboratory values				
TyG index	8.27 (8.08,8.43)	8.81 (8.67,8.97)	9.49 (9.21,9.72)	< 0.001
TG (mmol/L)	0.98 (0.82,1.14)	1.52 (1.24,1.86)	2.58 (2.12,3.34)	< 0.001
FBG (mmol/L)	5.00 (4.60,5.50)	5.30 (4.80,6.60)	6.05 (5.18,7.93)	< 0.001
HbA1c (%)	6.03 ± 0.68	6.28 ± 0.86	6.90 ± 1.39	< 0.001
CRP (mg/L)	3.46 ± 5.42	5.40 ± 12.00	3.00 ± 2.27	0.223
Serum creatinine (umol/L)	72.80 (61.30,82.75)	71.80 (59.00,81.10)	74.15 (63.23,86.73)	0.368
TC (mmol/L)	3.59 ± 0.91	3.83 ± 0.97	4.23 ± 1.67	0.001
LDL-C (mmol/L)	1.92 ± 0.83	2.26 ± 0.85	2.41 ± 0.99	0.003
LVEF (%)	62.73 ± 5.61	61.58 ± 6.51	61.43 ± 5.71	0.371
Coronary physiological parameters				
caFFR	0.95 (0.93,0.97)	0.96 (0.94,0.97)	0.95 (0.94,0.97)	0.227
caIMR	33.80 (29.40,38.75)	38.40 (30.13,46.63)	35.20 (29.08,43.53)	0.119
Cardiovascular medical therapy				
Aspirin, n (%)	49 (67.1)	53 (71.6)	53 (71.6)	0.790
Clopidogrel, n (%)	36 (49.3)	34 (45.9)	39 (52.7)	0.713
Statin, n (%)	67 (91.8)	61 (82.4)	64 (86.5)	0.243
ACEI/ARB, n (%)	38 (52.1)	32 (43.2)	30 (40.5)	0.342
Beta blocker, n (%)	34 (46.6)	35 (47.3)	34 (45.9)	0.986
CCB, n (%)	29 (39.7)	28 (37.8)	31 (41.9)	0.881

*TyG index* triglyceride-glucose index, *BMI* body mass index, *PCI* percutaneous coronary intervention, *CKD* chronic kidney disease, *CAD* coronary artery disease, *TG* triglyceride, *FBG* fasting blood glucose, *HbA1c* hemoglobin A1c, *CRP* C-reactive protein, *TC* total cholesterol, *LDL-C* low-density lipoprotein-cholesterol, *LVEF* left ventricular ejection fraction, *caFFR* coronary angiography-derived fractional flow reserve, *caIMR* coronary angiography-derived index of microcirculatory resistance, *ACEI/ARB* angiotensin-converting-enzyme inhibitor/angiotensin receptor blocker, *CCB* calcium channel blocker

### Association between the TyG index and CMD in CCS patients

**Fig. 3:**
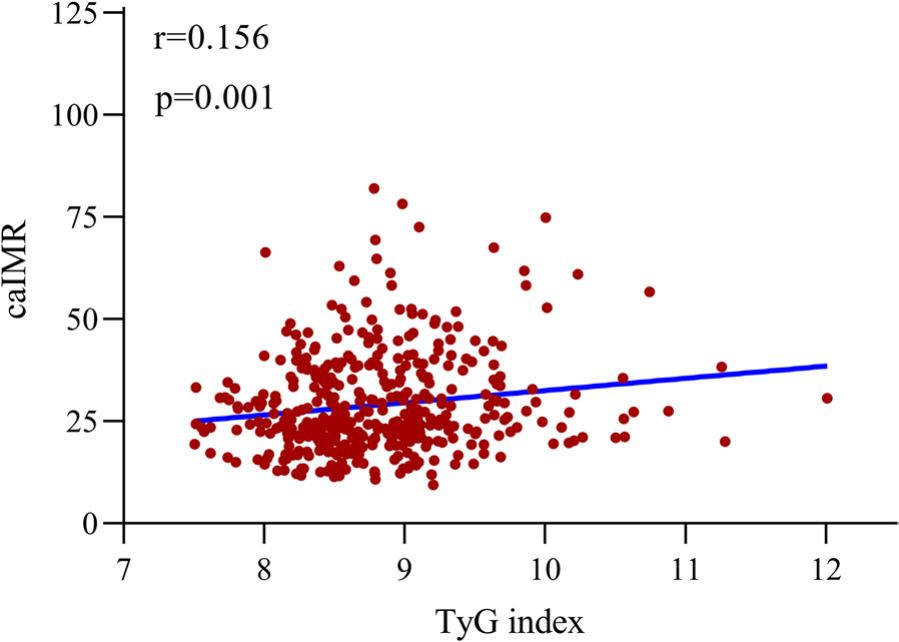
Correlation between TyG index and caIMR. *TyG index* triglyceride-glucose index, *caIMR* coronary angiography-derived index of microcirculatory resistance.

The association between the TyG index and CMD were shown in Table 3. Univariable logistic regression analysis indicated that TyG index, BMI, FBG, TG, TC, and the history of CAD, 3-vessel disease, PCI, and clopidogrel use were statistically correlated with CMD and that the risk of CMD increased by 62.9% for per unit increase in TyG index (OR, 1.629; 95% CI, 1.187–2.237; P = 0.003). After adjusting for confounding factors (the VIF of these variables were shown in Additional file 1: Table S1), the association between CMD and TyG index, BMI still maintained a significant difference and the risk of CMD increased by 43.6% per unit increase in TyG index (OR, 1.436; 95% CI, 1.014–2.034; P = 0.042). We further evaluated the association between TyG index and CMD in subgroup analysis. The results suggested that there was no interaction between TyG index and subgroups (gender, diabetes, CAD) for presence of CMD (All *p* for interaction > 0.05). However, the association between TyG index and CMD was stronger among females and non-diabetic population. (Additional file 1: Table S2). Figure 3 showed the correlation between the TyG index and caIMR. The results demonstrated that the TyG index correlated well with caIMR among CCS patients (r = 0.156, p = 0.001). The correlation between TyG index and caIMR existed in both CAD and non-CAD patients, and was also observed in females and non-diabetic patients, but not in males and diabetic patients (Additional file 1: Figure S1).

**Table 3:** Association between CMD and clinical risk factors

	Univariate analysis OR (95% CI)	P value	Multivariate analysis OR (95% CI)	P value
Age	0.991 (0.971–1.010)	0.352		
Male	0.979 (0.666–1.439)	0.915		
BMI	1.130 (1.060–1.204)	< 0.001	1.126 (1.051–1.206)	0.001
Heart rate	1.003 (0.987–1.020)	0.714		
Atrial fibrillation	0.640 (0.268–1.531)	0.316		
Smoking	0.988 (0.616–1.586)	0.962		
Hypertension	0.843 (0.569–1.248)	0.392		
PCI performed	0.624 (0.426–0.914)	0.015	1.209 (0.625–2.338)	0.573
Diabetes	1.328 (0.892–1.979)	0.162		
CKD	1.791 (0.836–3.835)	0.134		
CAD	0.510 (0.343–0.760)	0.001	0.557 (0.273–1.137)	0.108
1-vessel disease	0.803 (0.523–1.233)	0.316		
2-vessel disease	0.791 (0.500–1.250)	0.315		
3-vessel disease	0.540 (0.309–0.944)	0.031	0.764 (0.416–1.403)	0.385
TyG index	1.629 (1.187–2.237)	0.003	1.436 (1.014–2.034)	0.042
TG	1.204 (1.015–1.428)	0.033	–	
FBG	1.140(1.012–1.284)	0.031	–	
HbA1c	1.069 (0.905–1.263)	0.435		
CRP	1.005 (0.976–1.034)	0.749		
Serum creatinine	0.999 (0.990–1.009)	0.908		
TC	1.225 (1.012–1.483)	0.037	1.013 (0.687–1.493)	0.947
LDL-C	1.240 (0.997–1.542)	0.053	1.109 (0.714–1.723)	0.645
LVEF	1.003 (0.972–1.035)	0.840		
Aspirin	0.991 (0.655–1.498)	0.964		
Clopidogrel	0.568 (0.386–0.835)	0.004	0.702 (0.451–1.093)	0.117
Statin	0.662 (0.359–1.221)	0.187		
ACEI/ARB	0.936 (0.641–1.368)	0.733		
Beta blocker	0.916 (0.627–1.337)	0.648		
CCB	1.111 (0.753–1.639)	0.595		

*CMD* coronary microvascular dysfunction, *BMI* body mass index, *PCI* percutaneous coronary intervention, *CKD* chronic kidney disease, *CAD* coronary artery disease, *TyG index* triglyceride-glucose index, *TG* triglyceride, *FBG* fasting blood glucose, *HbA1c* hemoglobin A1c, *CRP* C-reactive protein, *TC* total cholesterol, *LDL-C* low-density lipoprotein-cholesterol, *LVEF* left ventricular ejection fraction, *ACEI/ARB* angiotensin-converting-enzyme inhibitor/angiotensin receptor blocker, *CCB* calcium channel blocker, *OR* odds ratio, *CI* confidence interval

### Clinical outcome of CMD patients based on the TyG index

**Fig. 4:**
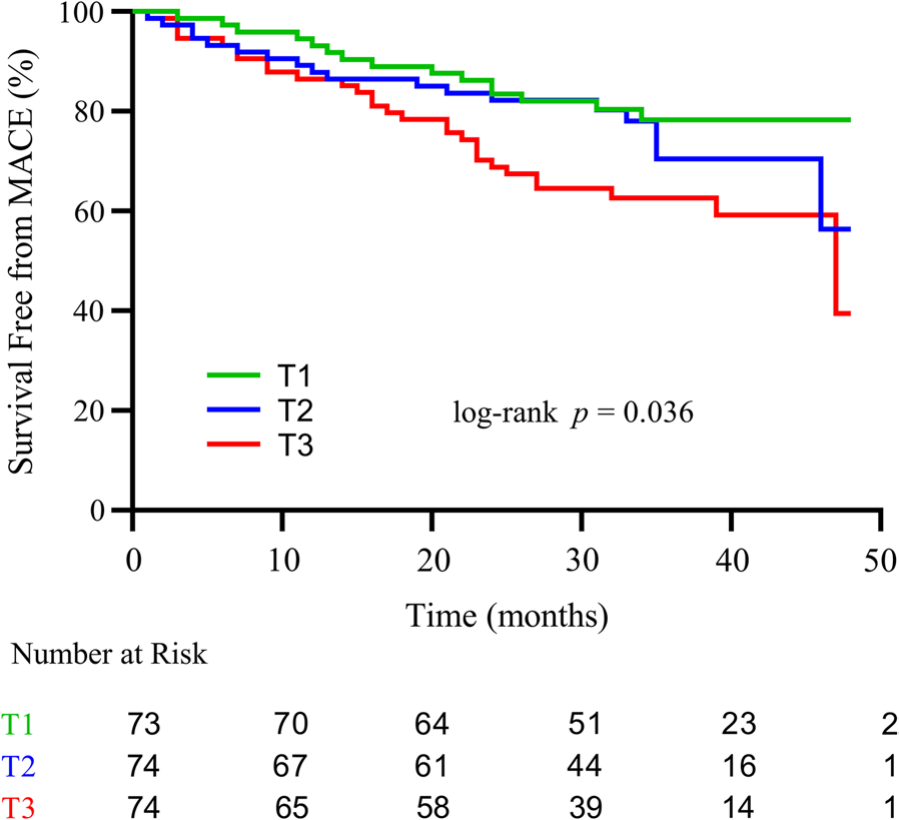
Kaplan–Meier survival curve for MACE in CMD patients with CCS according to TyG index tertiles. *MACE* major adverse cardiovascular events, *CMD* coronary microvascular dysfunction, *CCS* chronic coronary syndrome, *TyG index* triglyceride-glucose index, *T1* TyG index tertile 1, *T2* TyG index tertile 2, *T3* TyG index tertile 3.

The median follow-up duration was 35 months. Sixty-three (28.5%) MACE were recorded during the follow-up duration among the CMD population, including 4 (1.8%) cardiovascular death, 4 (1.8%) nonfatal MI, 18 (8.1%) heart failure, 15 (6.9%) nonfatal stroke, and 22 (10.0%) ischemia-driven revascularizations. Patients with higher TyG index (T3 group) had a higher rate of MACE as compared to lower TyG index patients (T1 and T2 groups) (39.2% vs. 20.5% vs. 25.7%; p = 0.035) (Table 4). Kaplan–Meier curve analysis showed that the risk of MACE in the T3 group was significantly higher than that in the T1 and T2 groups (log-rank P = 0.036) (Fig. 4).

**Table 4:** Clinical outcomes of CMD patients according to TyG index level

	TyG index level			P-value
	T1 (n = 73)	T2 (n = 74)	T3 (n = 74)	
MACE	15 (20.5)	19 (25.7)	29 (39.2)	0.035
Cardiovascular death	1 (1.4)	1 (1.4)	2 (2.7)	0.790
Nonfatal MI	1 (1.4)	0	3 (4.1)	0.114
Heart failure	3 (4.1)	8 (10.8)	7 (9.5)	0.292
Nonfatal stroke	5 (6.8)	2 (2.7)	8 (10.8)	0.146
Ischemia-driven revascularization	5 (6.8)	8 (10.8)	9 (12.2)	0.536

*CMD* coronary microvascular dysfunction, *TyG index* triglyceride-glucose index, *MACE* major adverse cardiovascular event, *MI* myocardial infarction

### Predictive factors of MACE

The results of the univariate and multivariate Cox regression analysis are displayed in Table 5. Univariable Cox regression analysis indicated that compared with the T1 group, the T3 group had a 2.157-fold risk of MACE (HR, 2.157; 95% CI 1.156–4.025; P = 0.016). After adjusting for potential covariates, including age, atrial fibrillation, diabetes, CKD, serum creatinine, LVEF, clopidogrel use, and ACEI/ARB use (the VIF of these variables were shown in Additional file 1: Table S3), the risk of MACE in the T3 group was still significantly higher when using the T1 group as the reference (HR, 2.132; 95% CI 1.066–4.261; P = 0.032). We then performed the subgroup analysis. Although the interactions of TyG index with CMD subgroups (gender, diabetes and CAD) were not significant, the association was stronger in CAD patients and patients without diabetes. In addition, a subgroup analysis according to LVEF was performed, after excluding the patients with LVEF < 50%, TyG index remained independent predictor of poor prognosis in multivariate models (Additional file 1: Table S4).

**Table 5:** Cox regression analysis for MACE of CMD patients

	Univariate analysis HR (95% CI)	P value	Multivariate analysis HR (95% CI)	P value
Age	1.024 (0.996–1.052)	0.090	1.027 (0.996–1.059)	0.085
Male	1.221 (0.737–2.025)	0.438		
BMI	0.999 (0.925–1.079)	0.981		
Heart rate	1.003 (0.982–1.025)	0.768		
Atrial fibrillation	2.443 (0.975–6.116)	0.057	1.256 (0.469–3.362)	0.650
Smoking	1.128 (0.621–2.049)	0.693		
Hypertension	1.367 (0.809–2.311)	0.243		
PCI performed	1.181 (0.719–1.938)	0.512		
Diabetes	1.828 (1.115–2.999)	0.017	1.287 (0.761–2.176)	0.346
CKD	1.926 (0.947–3.916)	0.070	0.873 (0.288–2.645)	0.810
CAD	1.369 (0.825–2.270)	0.224		
1- vessel disease	0.995 (0.557–1.778)	0.987		
2- vessel disease	1.174 (0.647–2.128)	0.597		
3- vessel disease	1.654 (0.816–3.353)	0.163		
TyG index	1.506 (1.085–2.090)	0.014	-	
TyG Tertiles				
T1	Reference		Reference	
T2	1.357 (0.689–2.674)	0.377	1.595 (0.785–3.241)	0.197
T3	2.157 (1.156–4.025)	0.016	2.132 (1.066–4.261)	0.032
TG	1.100 (0.960–1.261)	0.171		
FBG	1.141 (1.047–1.244)	0.003	-	
HbA1c	1.161 (0.946–1.425)	0.153		
CRP	0.990 (0.952–1.031)	0.633		
Serum creatinine	1.012 (1.001–1.024)	0.033	1.010 (0.991–1.028)	0.307
TC	1.054 (0.837–1.329)	0.654		
LDL-C	1.039 (0.794–1.359)	0.783		
LVEF	0.914 (0.884–0.944)	< 0.001	0.925 (0.890–0.961)	< 0.001
caFFR	0.011 (1 × 10^–6^-85.756)	0.324		
caIMR	0.998 (0.975–1.021)	0.856		
Aspirin	0.695 (0.416–1.162)	0.166		
Clopidogrel	1.606 (0.968–2.665)	0.067	1.341 (0.786–2.287)	0.282
Statin	1.243 (0.566–2.729)	0.588		
ACEI/ARB	1.521 (0.926–2.497)	0.097	0.997 (0.562–1.771)	0.993
Beta blocker	0.804 (0.488–1.325)	0.392		
CCB	1.048 (0.633–1.735)	0.855		

*MACE* major adverse cardiovascular event, *BMI* body mass index, *PCI* percutaneous coronary intervention, *CKD* chronic kidney disease, *CAD* coronary artery disease, *TyG index* triglyceride-glucose index, *TG* triglyceride, *FBG* fasting blood glucose, *HbA1c* hemoglobin A1c, *CRP* C-reactive protein, *TC* total cholesterol, *LDL-C* low-density lipoprotein-cholesterol, *LVEF* left ventricular ejection fraction, *caFFR* coronary angiography-derived fractional flow reserve, *caIMR* coronary angiography-derived index of microcirculatory resistance, *ACEI/ARB* angiotensin-converting-enzyme inhibitor/angiotensin receptor blocker, *CCB* calcium channel blocker, *HR* hazard ratio, *CI* confidence interval

## Discussion

The present study is the first to assess the association between the TyG index and the presence and prognosis of CMD in patients with CCS. The novel findings of our study included: (1) CMD patients had a significantly higher TyG index than those without CMD; (2) the TyG index significantly correlated with caIMR, and was an independent risk predictor for CMD in CCS patients; (3) high TyG index was independently associated with the risk of MACE in CMD patients with CCS.

The pathological mechanism of CCS is complex, and macrovascular disease of coronary arteries cannot fully explain the clinical manifestations and poor prognosis of CCS patients [1]. Many CCS patients without obvious coronary artery stenosis or after complete revascularization may still have typical anginal symptoms and poor prognosis [1, 3, 4]. Increasing attention has been paid to the adverse effects of CMD which may provide additional insights into risk modification for CCS patients [3]. CMD refers to the abnormal function and structure of coronary microvessels, which can result in insufficient blood supply to the myocardium [3]. Oxidative stress is considered to be a key mechanism of the development of CMD, which can cause impaired NO-mediated vasodilation, thereby promoting the progression of CMD [24, 25]. Coronary microcirculation impairments could cause myocardial ischemia, leading to clinical symptoms such as angina pectoris [3, 4]. A large number of studies have shown that CMD is common in patients with CCS and portends a poor prognosis, and is often accompanied by an impaired quality of life, mental status, and exercise capacity [2–4, 26]. Previous studies have suggested that coronary slow flow, a sign of CMD, was associated with decreased carotid blood flow velocity [27]. In recent years, more attention has been paid to the hazards of CMD. Rajai et al. reported that CMD was closely related to the development of cancer in patients with non-obstructive coronary artery disease [28]. The cerebral-coronary connection study suggested that CMD patients were more likely to have cognitive impairment [29]. The increasing recognition of the hazards associated with CMD has led to a significant rise in the importance of research on CMD. caIMR is a new method to detect CMD based on coronary angiography without pressure wire and hyperemic agents [19]. In previous studies, caIMR (cut-off 25U) showed a good correlation with invasive IMR which is a specific tool to detect CMD [6, 19]. Recently, several studies have shown that CMD diagnosed by caIMR was associated with poor prognosis. Choi et al. indicated that ST-segment elevation myocardial infarction (STEMI) patients with CMD assessed by caIMR showed an increased risk of cardiac death or heart failure readmission [30]. Abdu et al. reported that CMD assessed by caIMR was closely related to the poor prognosis of MINOCA patients [7]. Neng et al. found that CMD diagnosed by caIMR was a strong predictor for cardiac death and heart failure in CAD patients [6]. We recently found that CMD measured by caIMR was common and was a reliable predictor of clinical outcomes in diabetic patients with CCS [31]. Based on this, CMD is clearly associated with poor prognosis, and the identification of predictive factors associated with CMD may be necessary for prompt risk stratification.

Type 2 diabetes mellitus (T2DM) is characterized by IR, which has been identified as a CVD risk factor and as a contributor to the development of CMD [10, 11, 32]. IR can decrease the production of vasoprotective nitric oxide (NO) by reducing the activity of the endothelial NO synthase leading to CMD [33]. In addition, hyperinsulinemia can also promote oxidative stress and the release of pro-inflammatory cytokines by increasing free fatty acids, reactive oxygen species (ROS) production, and dyslipidemia, thus promoting CMD [11]. Recently, A small study also suggested that CMD is significantly associated with the severity of IR during acute hyperglycemia [10]. Currently, IR detection methods are expensive, time-consuming, difficult to use in clinical practice, and have significant bias [14]. The TyG index, as the product of FBG and fasting TG, is a novel index that has been suggested as a simple and reliable surrogate of IR and is not affected by insulin treatment [12–14]. A high TyG index was associated with a variety of CVD such as symptomatic CAD, arterial stiffness, coronary artery calcification, in-stent restenosis, and multi-vessel CAD [14, 34–37]. Moreover, TyG index was also associated with microvascular diseases including microangiopathies, nephric microvascular damage, microalbuminuria, and slow coronary flow [18, 38–40]; these findings suggest that there may be an association between the TyG index and the risk of CMD. these findings suggested that TyG index might be associated with the risk of CMD. In the present study, we found that CMD patients had significantly higher TyG index than non-CMD patients; we also found that the TyG index was an independent predictor of CMD in CCS patients. The findings of our study are consistent with previous reports suggesting an indirect association between insulin resistance (IR) and CMD. This highlights the need for interventions to prevent the occurrence of CMD in CCS patients by targeting IR. In addition, our study also revealed that the link between TyG index and CMD was stronger in females and non-diabetic patients. Similarly, the association between TyG index and caIMR was more prominent in these subgroups. These findings are in line with previous research, which has suggested that the impact of TyG index may be more pronounced in females and non-diabetic individuals [16, 17, 41, 42]. However, it is important to note that a direct cause-effect role of TyG index with CMD is less certain and further studies are needed to fully elucidate the underlying mechanisms.

A large number of studies have shown that the TyG index can predict adverse outcomes in patients with CVD [14, 16, 17, 43, 44]. Wang et al. reported that the TyG index could predict the risk of MACE in ACS patients with diabetes [45]. In patients with CAD, the TyG index was associated with T2DM and was an independent predictor of future cardiovascular events [46, 47]. Zhao et al. found that the TyG index in combination with microstructural optical coherence tomography (OCT) characteristics of culprit lesions could support risk stratification in STEMI patients [44]. Several studies have shown that the TyG index can also be used in the risk stratification of patients with premature CAD or chronic total occlusion [16, 17]. However, the prognostic value of TyG index in the CMD population is currently unknown, and identifying high-risk patients through risk stratification of CMD is of great clinical significance. Consistent with previous studies, this study showed that TyG index was not only associated with the presence of CMD but also remained strongly correlated with the risk of MACE in CCS patients with CMD. Multivariate cox regression analysis also showed that the highest tertile of the TyG index remained significantly correlated with worse clinical outcomes even after adjustment of other confounding risk factors. This underlines the importance of lipid and glucose metabolism, as revealed by the TyG index, in the pathogenic mechanism of CMD. Subgroup analysis showed that there was no interaction between CAD and TyG index for prognosis of CMD patients and TyG index was still associated with the occurrence of MACE in CMD patients with CAD. After excluding patients with LVEF < 50%, the correlation between TyG index and MACE in CMD patients remained (Additional file 1: Table S4). These results further support the prognostic value of TyG index for CMD patients, rather than the presence of previous cardiovascular diseases that leads to poor prognosis. Our results provided significant clinical evidence for the predictive value of the TyG index in CMD patients for the occurrence of MACE. The TyG index is a simple and easily obtained measure, which is not affected by insulin therapy. Therefore, it has better clinical applicability in the risk stratification of CMD patients. Targeting IR in CCS patients may be beneficial in the prevention and treatment of CMD, which needs further confirmation.

### Study limitations

There are some potential limitations in our study. First, this is a single-center retrospective observational study, and the sample size is relatively small, therefore, our study does not confirm a causal relationship between elevated TyG index and the development of CMD. Further large-scale, prospective, and multi-center studies are needed to validate the results of the present study. Second, as a novel surrogate technique, caIMR is operator-dependent to some extent. In our study, participants did not receive invasive IMR assessment for CMD. Third, the patients’ TG and FBG levels were only tested at admission and their fluctuations were not measured during the follow-up; therefore, it was unknown whether the TyG index changed during the follow-up. Finally, our study mainly focused on the TyG index and lacked other glucose-related variables such as diabetic drugs, HOMA-IR, and insulin level. Despite these limitations, our study is important for clinical practice as it is the first to investigate the relationship between the TyG index and the presence and prognosis of CMD in CCS patients.

## Conclusion

This study demonstrates for the first time that the increased TyG index is significantly associated with a higher risk of CMD in CCS patients, and it is a strong independent predictor for MACE among CMD patients with CCS. This conclusion suggests that the TyG index has important clinical significance for the early prevention and risk stratification of CMD.

## Supplementary Information

**Additional file1: Figure S1.** Correlation between TyG index and caIMR in various subgroups. *TyG index* triglyceride-glucose index, *caIMR* coronary angiography-derived index of microcirculatory resistance, *DM* diabetes mellitus, *CAD* coronary artery disease.

## Data Availability

The data analyzed in this study can be obtained from the corresponding author with a reasonable request.

## Abbreviations

CMD: Coronary microvascular dysfunction
CCS: Chronic coronary syndrome
TyG index: Triglyceride-glucose index
caIMR: Coronary angiography‑derived index of microvascular resistance
MACE: Major adverse cardiac events
CAD: Coronary artery disease
CVD: Cardiovascular disease
ACS: Acute coronary syndrome
HFpEF: Heart failure with preserved ejection fraction
MINOCA: Myocardial infarction with nonobstructive coronary arteries
IR: Insulin resistance
FBG: Fasting blood glycemia
TG: Triglycerides
CAG: Coronary angiography
MI: Myocardial infarction
CABG: Post coronary artery bypass graft surgery
LVEF: Left ventricular ejection fraction
HbA1c: Hemoglobin A1c
TC: Total cholesterol
LDL-C: Low-density lipoprotein-C
CRP: C-reactive protein
PCI: Percutaneous coronary intervention
SBP: Systolic blood pressure
DBP: Diastolic blood pressure
BMI: Body mass index
CKD: Chronic kidney disease
VIF: Variance inflation factor
STEMI: ST-segment elevation myocardial infarction
T2DM: Type 2 diabetes mellitus
NO: Nitric oxide
ROS: Reactive oxygen species
OCT: Optical coherence tomography
