# Supplementary material for: Association between the triglyceride-glucose index and the presence and prognosis of coronary microvascular dysfunction in patients with chronic coronary syndrome
Source: Cardiovasc Diabetol. 2023 May 13;22:113. doi: 10.1186/s12933-023-01846-z (PMC10183136; doi:10.1186/s12933-023-01846-z)
Supplement: Supplementary file 1 — Additional file1: Figure S1. Correlation between TyG index and caIMR in various subgroups. TyG index triglyceride-glucose index, caIMR coronary angiography-derived index of microcirculatory resistance, DM diabetes mellitus, CAD coronary artery disease. [file 12933_2023_1846_MOESM1_ESM.zip › Additional file 1stables.docx]

**Table S1.** Collinearity diagnostics for the variables included in multivariate logistic regression analysis

| Variables | Tolerance | VIF |
| --- | --- | --- |
| TC | 0.261 | 3.828 |
| LDL-C | 0.268 | 3.730 |
| BMI | 0.930 | 1.076 |
| PCI performed | 0.360 | 2.779 |
| CAD | 0.326 | 3.067 |
| 3-vessel disease | 0.895 | 1.117 |
| TyG index | 0.830 | 1.204 |
| Clopidogrel | 0.810 | 1.235 |

*VIF* variance inflation factor, *TC* total cholesterol, *LDL-C* low-density lipoprotein-cholesterol, *BMI* body mass index, *PCI* percutaneous coronary intervention, *CAD* coronary artery disease, *TyG index* triglyceride-glucose index.

**Table S2.** Subgroup analysis between the TyG index and CMD

|  | Adjusted OR (95% CI) | P value | P for interaction |
| --- | --- | --- | --- |
| **Gender** |  |  | 0.916 |
| Male |  |  |  |
| TyG index | 1.341 (0.869-2.070) | 0.185 |  |
| Female |  |  |  |
| TyG index | 1.864 (1.074-3.236) | 0.027 |  |
| **Diabetes** |  |  | 0.135 |
| Yes |  |  |  |
| TyG index | 1.263 (0.807-1.977) | 0.306 |  |
| No |  |  |  |
| TyG index | 1.767 (1.047-2.982) | 0.033 |  |
| **CAD** |  |  | 0.179 |
| Yes |  |  |  |
| TyG index | 1.512 (0.999-2.289) | 0.051 |  |
| No |  |  |  |
| TyG index | 1.616 (0.869-3.006) | 0.129 |  |

*TyG index* triglyceride-glucose index, *CMD* coronary microvascular dysfunction, *CAD* coronary artery disease, *OR* odds ratio, *CI* confidence interval.

**Table S3.** Collinearity diagnostics for the variables included in multivariate Cox regression analysis

| Variables | Tolerance | VIF |
| --- | --- | --- |
| Age | 0.904 | 1.106 |
| Atrial fibrillation | 0.925 | 1.081 |
| Diabetes | 0.881 | 1.135 |
| CKD | 0.535 | 1.869 |
| TyG index tertiles | 0.864 | 1.158 |
| Serum creatinine | 0.541 | 1.850 |
| LVEF | 0.895 | 1.117 |
| Clopidogrel | 0.941 | 1.062 |
| ACEI/ARB | 0.895 | 1.117 |

*VIF* variance inflation factor, *CKD* chronic kidney disease, *TyG index* triglyceride-glucose index, *LVEF* left ventricular ejection fraction, *ACEI/ARB* angiotensin-converting-enzyme inhibitor/angiotensin receptor blocker.

**Table S4.** Subgroup analysis between the TyG index tertiles and MACE in CMD patients

|  | Group | Adjusted HR (95% CI) | P value | P for interaction |
| --- | --- | --- | --- | --- |
| **Gender** |  |  |  | 0.300 |
| Male | T3 | 2.342 (0.987-5.558) | 0.054 |  |
| Female | T3 | 1.782 (0.599-5.303) | 0.299 |  |
| **Diabetes** |  |  |  | 0.174 |
| Yes | T3 | 1.037 (0.458-2.349) | 0.930 |  |
| No | T3 | 2.640 (1.014-6.878) | 0.047 |  |
| **CAD** |  |  |  | 0.115 |
| Yes | T3 | 3.159 (1.322-7.548) | 0.010 |  |
| No | T3 | 1.151 (0.427-3.106) | 0.781 |  |
| **LVEF** |  |  |  | 0.125 |
| LVEF≥50% | T3 | 2.552 (1.210-5.383) | 0.014 |  |

*TyG index* triglyceride-glucose index, *MACE* major adverse cardiovascular event, *CMD* coronary microvascular dysfunction, *T3* TyG index tertile 3, *CAD* coronary artery disease, *LVEF* left ventricular ejection fraction, *HR* hazard ratio, *CI* confidence interval.
