# Supplementary figures and images for: Association between the triglyceride-glucose index and the presence and prognosis of coronary microvascular dysfunction in patients with chronic coronary syndrome
Source: Cardiovasc Diabetol. 2023 May 13;22:113. doi: 10.1186/s12933-023-01846-z (PMC10183136; doi:10.1186/s12933-023-01846-z)

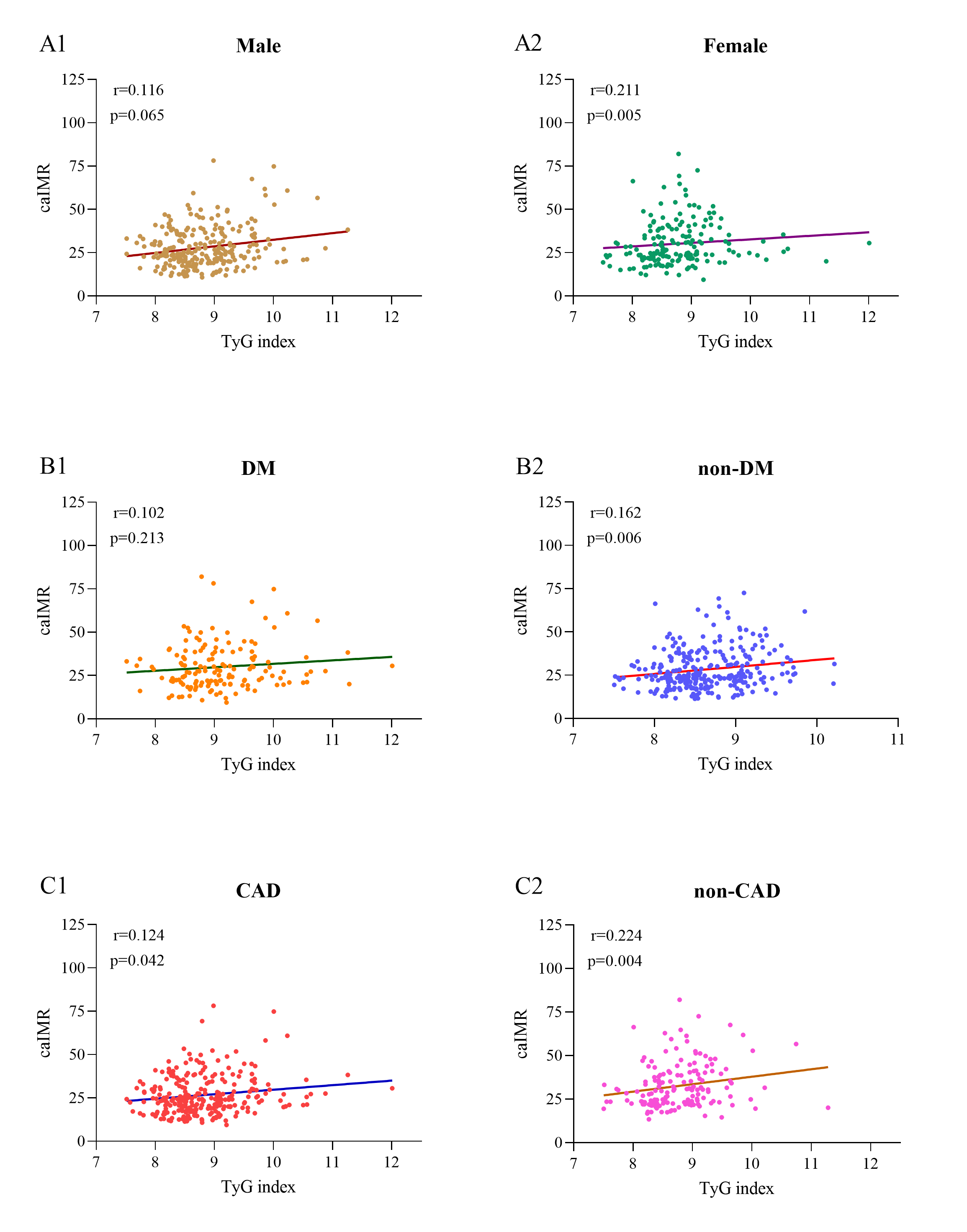

Supplement: Supplementary file 1 — Additional file1: Figure S1. Correlation between TyG index and caIMR in various subgroups. TyG index triglyceride-glucose index, caIMR coronary angiography-derived index of microcirculatory resistance, DM diabetes mellitus, CAD coronary artery disease. [file 12933_2023_1846_MOESM1_ESM.zip › Additional file 1sFigure S1.tif]
